# 2-Butanol and Butanone Production in *Saccharomyces cerevisiae* through Combination of a B_12_ Dependent Dehydratase and a Secondary Alcohol Dehydrogenase Using a TEV-Based Expression System

**DOI:** 10.1371/journal.pone.0102774

**Published:** 2014-07-23

**Authors:** Payam Ghiaci, Joakim Norbeck, Christer Larsson

**Affiliations:** Department of Chemical and Biological Engineering, System and Synthetic Biology, Chalmers University of Technology, Gothenburg, Sweden; University of Nottingham, United Kingdom

## Abstract

2-Butanol and its chemical precursor butanone (methyl ethyl ketone – MEK) are chemicals with potential uses as biofuels and biocommodity chemicals. In order to produce 2-butanol, we have demonstrated the utility of using a TEV-protease based expression system to achieve equimolar expression of the individual subunits of the two protein complexes involved in the B_12_-dependent dehydratase step (from the pdu-operon of *Lactobacillus reuterii*), which catalyze the conversion of *meso*-2,3-butanediol to butanone. We have furthermore identified a NADH dependent secondary alcohol dehydrogenase (Sadh from *Gordonia* sp.) able to catalyze the subsequent conversion of butanone to 2-butanol. A final concentration of 4±0.2 mg/L 2-butanol and 2±0.1 mg/L of butanone was found. A key factor for the production of 2-butanol was the availability of NADH, which was achieved by growing cells lacking the *GPD1* and *GPD2* isogenes under anaerobic conditions.

## Introduction

Researchers have been contemplating options for substituting fossil fuels for more than a decade. Biofuels made by microorganisms have been among the options with ethanol, fatty acid methyl esters (biodiesel) and different butanol isomers (1-butanol, 2-butanol and iso-butanol) being among the more prominent. Butanol as a fuel, compared to ethanol, has greater energy content while having lower vapour pressure and containing less water [1]. The three named isomers of butanol have different physical and chemical characteristics but all of them are considered as high value components either as chemicals or fuel. Various biological routes have been suggested for each of the three butanol isomers, such as amino acid pathways redirection for 1-butanol and isobutanol [2]–[4] and exploitation of the 1-butanol synthesis pathway in *clostridium* species [5]–[7]. 2-Butanol production can theoretically be introduced in yeast by a two-step conversion as found e.g. in *Lactobacillus* species; dehydration of 2,3-butanediol to butanone and hydrogenation of butanone to 2-butanol [8]. A fourth form of butanol (tert-butanol) exists, but it is solid at room temperature and it is furthermore not known to be produced biologically in any organism, hence it cannot be regarded as a biofuel. 2-Butanol, in particular, holds superior fuel characteristics among the butanol isomers. 2-butanol has the highest research octane number and motor octane number among the isomers (110 and 93 respectively) while they all have rather similar heating values (1-butanol: 27, 2-butanol: 26.8, iso-butanol: 26.6 MJ/L) [9].

**Figure 1:**
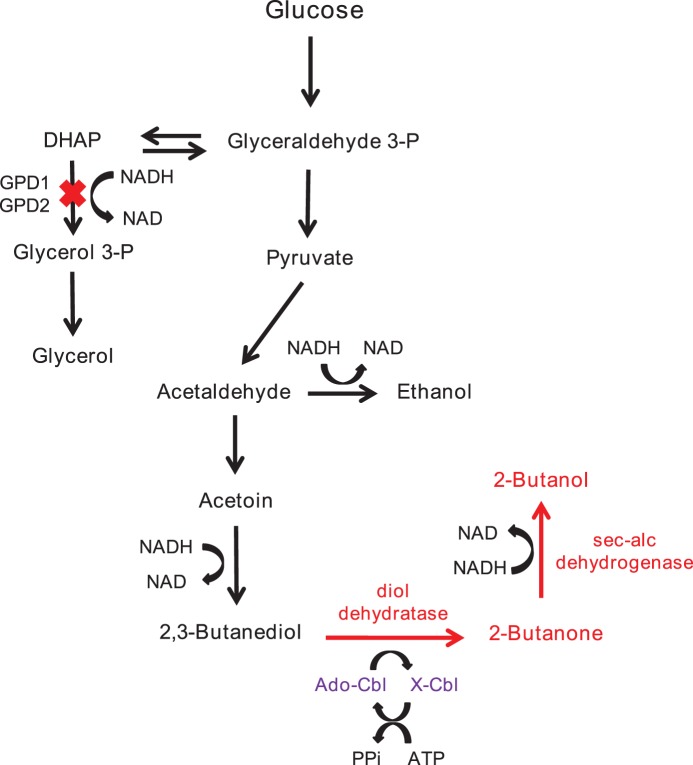
Overview of the pathway introduced to *S. cerevisiae* for 2-butanol production. The heterologous proteins expressed are marked in red. In violet is the Ado-Cbl recycling system. X-Cbl is an unidentified complex of cobalamine made due to inactivation [34].

The main aim of this project was to evaluate the possibility of producing 2-butanol from *meso*-2,3-butanediol in *Saccharomyces cerevisiae* (Figure 1), as a safe microorganism with a long history of industrial usage. 2,3-butanediol is known to be produced in *S. cerevisiae*, in low amounts, and there are a number of reports on its production improvement through introducing heterologous enzymes and blocking of competing pathways [10], [11].

Despite the fact that there are a lot of genetic tools available to engineer *S. cerevisiae*, a common issue in many cases of metabolic engineering (e.g. for biofuel production) is the necessity to simultaneously express multiple genes in order to make efficient cell factories. This is important particularly in cases of proteins with multiple subunits, where proper expression of each subunit is required in order to have a functional protein complex. A conventional way to express multiple genes simultaneously is to use plasmid vectors, each holding a gene of interest. However, this method loses its efficiency with increased numbers of vectors, mainly due to the lack of marker genes and suitable promoter and terminator sequences, and the risk of plasmid recombination. Another option is to express multiple genes in a single vector, where each gene has its own promoter and terminator. However, normal plasmid vectors should not be bigger than 10–15 kb, which rapidly puts a limit on the number of genes that can be inserted on each plasmid. There are a number of approaches available in *S. cerevisiae* to circumvent this problem, for example several studies have used yeast artificial chromosomes [12]–[17] and multiple loci genomic integrations in combination with marker recycling. An alternative expression system for multiple proteins is the polyprotein proteolytic process in tobacco etch viruses (TEVs) [18], [19]. In this case, several proteins are expressed as a single open reading frame (i.e. a poly-protein), separated from one another by TEV-protease cleavage sites. Subsequent expression of a TEV protease, either separately or as part of the poly-protein, causes cleavage of the poly-protein into individual proteins [20]. The advantages of this method are that all proteins will be expressed at an equimolar level and also at the same intracellular localization. Furthermore, an important feature of this system is that the use of regulatory sequences can be minimized, thereby allowing addition of more genes on each plasmid. This method has been successfully applied in plants [21], *E. coli* and mammalian cells [18] but to our knowledge not in *S. cerevisiae*. The TEV protease itself has in several cases been used for protein modification in *S. cerevisiae* without known adverse effects on the host cell [22], [23].

In this study, a TEV-cleavage based system was used to express all the three subunits of a B_12_-dependent diol dehydratase (pduC, pduD and pduE) from *Lactobacillus reuteri* and its two-subunit activating enzyme, (pduG and pduH) [24], in order to produce 2-butanol. The mature dehydratase enzyme converted *meso*-2,3-butanediol to butanone which was subsequently reduced to 2-butanol through expression of a secondary alcohol dehydrogenase (Sadh – encoded by *ADH2* gene) from *Gordonia* sp. strain TY-5 [25]. To the knowledge of authors, 2-butanol production has not been reported previously in *S. cerevisiae*.

## Materials and Methods

### Strain, medium and cultivation conditions

The host strains used for heterologous protein expression were *Δgpd1,2* double mutant *S. cerevisiae in W303-1A* background (*YSH6.142-3D*) [26] and wild type yeast in the *BY4742* background. A List of all the strains used in this study is provided in Table 1. The cells were grown in CBS medium (pH 5) with the following constituents: 20 g/L Glucose, 7.5 g/L (NH_4_)_2_SO_4_, 3.5 g/L KH_2_PO_4_, 0.744 g/L MgSO_4_.7H_2_O, 0.03 g/L EDTA, 0.009 g/L CaCl2.2H2O, 0.009 g/L ZnSO_4_.7H_2_O, 0.006 g/L FeSO_4_.7H_2_O, 0.002 g/L H_3_BO_3_, 0.0016 g/L MnCl_2_.2H_2_O, 0.0008 g/L Na_2_MoO_4_.2H_2_O, 0.0006 g/L CoCl_2_.6H_2_O, 0.0006 g/L CuSO_4_.5H_2_O, 0.0002 g/L KI, 0.2 mg/L p-Aminobenzoic acid, 0.05 mg/L D-Biotin, 1 mg/L Nicotinic acid, 1 mg/L Ca-pantothenate, 1 mg/L Pyridoxine.HCl, 1 mg/L Thiamine.HCl, 25 mg/L m-Inositol, 50 µl Antifoam Sigma A8436. For anaerobic cultures (in the case of *Δgpd1,2* strain), 420 mg/L Tween 80 and 10 mg/L Ergosterol were added to the medium.

**Table 1:** List of strains and DNA constructs used in this study.

	Description	Reference
**Strain**		
BY4742	*MATα his3Δ1 leu2Δ0 lys2Δ0 ura3Δ0*	[35]
yPG-1	BY4742 *p316-CDE, p315-SADH, pduGH integrated*	This study
YSH6.142-3D	W303-1A *MATα gpd1Δ::TRP1 gpd2Δ::URA3*	[26]
yPG-2	YSH6.142-3D *p315-SADH*	This study
yPG-3	YSH6.142-3D *p313-CDE, p315-SADH, pduGH integrated*	This study
yPG-4	YSH6.142-3D *p313-CDE-nt, p315-SADH, pduGH integrated*	This study
**Plasmid**		
pRS313	*HIS3*	[36]
pRS315	*LEU2*	[36]
pRS316	*URA3*	[36]
p313-CDE	pRS313+*pduC*, *pduD*, *pduE HIS3* P*_TPI1_*	This study
p313-CDE-nt	pRS313+*pduC*, *pduD*, *pduE* without TEV *HIS3* P*_TPI1_*	This study
p316-CDE	pRS316+*pduC*, *pduD*, *pduE URA3* P*_TPI1_*	This study
p315-SADH	pRS315+*SADH LEU2* P*_TDH3_*	This study
**Genome integrated const.**		
pUC57-pduGH	*pduGH*-construct and *KanMX4* flanked by homology domains to *DAK2* downstream sequence.	This study

In the case of *Δgpd1,2* strain, cells were cultivated aerobically overnight in 100 ml shake flasks (20 ml of culture at 30°C and 150 rpm). After reaching the OD_600_>1, a mixture of 1 g/L Adenosylcobalamine (Ado-Cbl/Coenzyme B_12_), substrate (3 g/L *meso*-2,3-Butanediol, 3 g/L racemic 2,3-Butanediol or 2 g/L Acetoin), 420 mg/L Tween 80 and 10 mg/L Ergosterol was added to the medium and anaerobic condition was applied by purging N_2_ for 30 minutes and attachment of anaerobic loops (i.e. a U-shape water lock glass tube, used to avoid O_2_ diffusion). In the case of the strain *BY4742,* cells were only grown aerobically with the substrate and Ado-Cbl being added to the medium from the start (20 ml of culture at 30°C and 150 rpm). All the strains were stored at −80°C with 20% glycerol.

### Plasmid construction and genomic DNA integration

**Figure 2:**
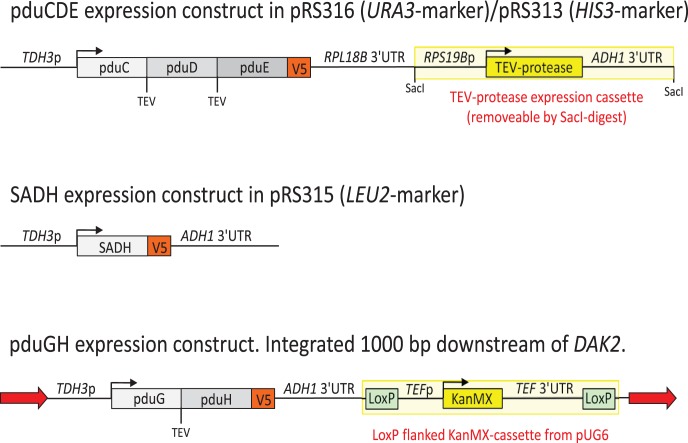
Constructs required for B12 dependent 2-butanol production in yeast. Depiction of the 4 constructs made for pduCDE, pduGH and SADH respectively. The pduCDE construct was made with and without the *SacI* fragment containing the TEV-protease.

Four sets of different DNA constructions were used, as outlined in Figure 2. The genes used in the cloning process were ordered as synthetic fragments (Genscript). *pduC*, *pduD* and *pduE*, as three subunits of *Lactobacillus reuteri* diol dehydratase together with a TEV protease were ligated into pRS316 and pRS313 plasmids as a *XhoI-NotI* fragment. A *NotI* site was introduced into the multiple cloning site of pRS316. Two TEV cleavage sites were inserted between the three *Lactobacillus* genes for *in vivo* processing of the pro-protein. *pduE* was tagged with a V5 epitope for later detection in Western blot. A TEV protease free version of these constructs was made by religation of a *SacI* cut *pduCDE-TEV* plasmid. A codon optimized secondary alcohol dehydrogenase (Sadh) from *Gordonia* sp. [25] was ligated into pRS315 plasmid as a *XhoI-SacI* fragment. Codon optimization was done through the webpage www.ecorbio.com. Codon optimized versions of the *pduG* and *pduH* open reading frames from *L. reuterii*, coding for two subunits of diol dehydratase activator, were introduced into an integrative construct, separated by a TEV-cleavage site. The integrative construct was transformed into the *Δgpd1,2* and *BY4742* strains by regular LiAc high efficiency transformation. *pduGH* was integrated into the genome 1000 bp downstream of the *DAK2* stop codon. The *pduH* subunit was also tagged with a V5 epitope for later detection in Western blot. *pduCDE* was expressed under control of a *TPI1* promoter and *pduGH* and *SADH* were expressed under a *TDH3* promoter. The TEV protease was under control of a *RPS19B* promotor which is adjacent to the *RPL18B* 3′UTR used to terminate the *pduCDE* transcription. All the DNA constructs are listed in Table 1 and the sequences of all the constructs are provided as Text S1.

### Protein extraction

Cultivated cells from 5 ml of culture at an OD_600_≈2 (grown in CBS medium and in presence of 2,3-butanediol and Ado-Cbl) were centrifuged. The pellet was washed with ice-cold Tris-Buffered Saline (TBS) buffer and resuspended in 1 ml of the same buffer. 1 µl protease inhibitor (Halt Protease Inhibitor Cocktail, EDTA-free 100X Thermo Scientific) and 1 g sterile glass bead was added. The mixture was shaken vigorously by Fast Prep (30 seconds at speed 6 - MP Biomedicals Solon) for 4 times. There was a 1 minute rest on ice between each shaking. The sample was centrifuged for 15 minutes at 14000 rpm and the supernatant was used as a crude protein extract.

### SDS-PAGE and western blot

10 µl of crude extract was mixed with 10 µl of 2X LDS sample buffer (NuPAGE Invitrogen) and 2 µl of 10x sample reducing agent (NuPAGE Invitrogen) was added. Sample and 10 µl of protein ladder (Spectra Multicolor Broad Range Protein Ladder – Thermo Scientific) were boiled for 5 minutes at 95°C. A precast MINI-PROTEAN TGX Gel (4–20% - Bio-Rad) was used for SDS-PAGE. The gel was run for 1.5 hour at 150 V. Proteins were transferred from the gel to a PVDF membrane (Bio-Rad) using a semi-dry transfer unit (TE77 PWR – Hoefer, 75 minutes, 50 mA). A transfer stack, composed of a three-layer blotting-paper (cut slightly smaller than the gel), a PVDF membrane, the gel and a second three-layer blotting-paper, was made. Each blotting paper was saturated with transfer buffer (25 mM Tris, 190 mM Glycine, 20% Methanol, 0.1% SDS – pH 8.3) and the PVDF membrane was pre-wet with methanol. After transferring proteins, the membrane was rinsed with water and stained with Ponceau S solution (Sigma) to check the quality of transfer. Subsequently, the membrane was washed three times with TBST buffer (20 mM Tris pH 7.5, 150 mM NaCl, 0.1% Tween 20), blocked in blocking buffer (3% bovine serum albumin, BSA, in TBST) for 1 hour at room temperature and incubated overnight in a primary antibody solution (anti-V5 antibody (Invitrogen R960-25) - 5000x diluted) at 4°C. After washing in TBST (3 times, each time for 5 minutes), goat anti-mouse IgG-HRP (Santa Cruz Biotechnology - 5000x diluted) was used as a secondary antibody in which the membrane was incubated for 1 hour at room temperature. The membrane was again washed in TBST (3 times, each time for 5 minutes). ECL Prime Western Blotting Detection Reagent (GE Healthcare) was used as chemiluminescent substrate. The membrane was incubated in a solution of equal amount of Solution A (Luminol enhancer solution) and Solution B (peroxide solution) for 5 minutes at room temperature (not exposed to light).

### Analysis of substrate and metabolites

2-Butanol, Butanone, Glycerol, Glucose, Acetate and Ethanol were analyzed by HPLC (Ultimate 3000, Dionex). A standard curve was made for each metabolite within the following concentration ranges: 2-Butanol and Butanone (1–20 mg/L), Glucose (1–20 g/L), Ethanol (1–15 g/L), Glycerol (0.1–2 g/L), Acetate (0.1–2 g/L). The column used was an Aminex HPX-87H column (300×7.8 mm - Bio-Rad) connected to a VWD-3100 detector (Thermo Scientific Dionex) with 5 mM H_2_SO_4_ as the eluent and a flow of 0.6 ml/min. The column was run at 45°C. All samples were analyzed in duplicates.

All results presented were performed as two independent biological replicates.

## Results and Discussion

The aim of the present work was to investigate the possibility of producing 2-butanol in yeast through the B_12_-dependent diol dehydratase system as outlined in Figure 1. This pathway relies on the two-step enzymatic conversion of 2,3-butanediol to 2-butanol, via butanone. The first step is catalyzed by a B_12_-dependent diol dehydratase while the second step is catalyzed by a secondary alcohol dehydrogenase (Sadh).

For the dehydrogenation-step we evaluated if the *ADH2* gene from *Gordonia* sp. [25], encoding one of three NADH-dependent secondary alcohol dehydrogenases in this organism, could be a suitable enzyme for butanone to 2-butanol conversion. The gene *ADH2* will henceforth be referred to as *SADH*. The Sadh was chosen because it has minor activity with primary alcohols, combined with a good activity for butanone [25]. We therefore cloned the *Gordonia SADH* as a codon optimized V5-epitope tagged version after the *TDH3*-promotor in a pRS315 (*LEU2*-marker) vector. The Sadh was then expressed in strains carrying deletions in *GPD1* and *GPD2* (strain *YSH6.142-3D*). Growth of a *Δgpd1,2* strain transformed with the SADH-plasmid (strain *yPG-2*) and fed with 0.8 g/L 2-butanone under aerobic conditions, did not lead to any 2-butanol production. However, supplying the cells with 2 g/L 2-butanol led to production of 1.5 g/L 2-butanone. A reasonable explanation for the favoured reverse reaction is that the NADH/NAD ratio is much lower under aerobic compared to anaerobic conditions [27]. Anaerobically, *S. cerevisiae* is in need of an endogenous electron acceptor to restore redox balance; a role which is played by glycerol production [28]. Incapability of the *Δgpd1,2* strain in producing glycerol results in a surplus of NADH which under aerobic conditions is oxidized via respiration but under anaerobic conditions deters the cell from growing since the cell does not have any alternative to oxidize the NADH. Therefore, in order to ensure an ample supply of NADH, the Sadh expressing *Δgpd1,2* strain of *S. cerevisiae (yPG-2)* was cultivated under anaerobic conditions which resulted in the formation of 42 mg/L 2-butanol from 2 g/L 2-butanone after 72 hours. A further increase was seen upon longer incubation (data not shown). We concluded that in the presence of high NADH-concentrations, the SADH from *Gordonia* sp. can perform the desired reaction to convert butanone to 2-butanol.

**Figure 3:**
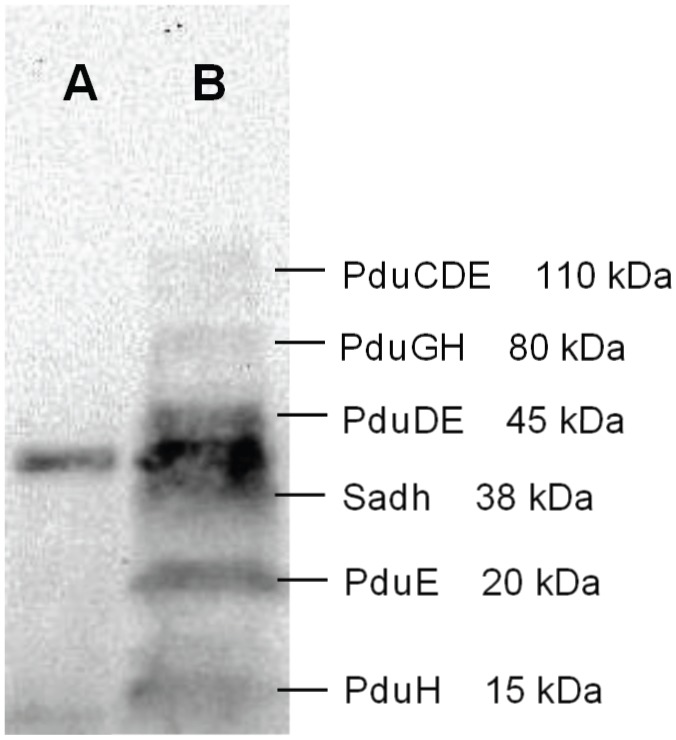
Verification of the TEV-strategy by western analysis. Western blot analysis of the control strain where no heterologous protein of 2-butanol pathway was expressed (strain *YSH6.142-3D* - Column A) and the strain with the entire set for TEV expression system (pduCDE+pduGH) together with the secondary alcohol dehydrogenase (Sadh) (strain *yPG-3* - Column B). Data is shown for *W303 Δgpd1,2* background.

Our next aim was to express a functional diol-dehydratase in *S. cerevisiae*, which is a more complex task than the Sadh. This step of the pathway relies on equimolar expression of a three subunit diol dehydratase complex (pduCDE) and a likewise equimolar amount of both proteins in a two subunit activating enzyme (pduGH). We decided to try an approach in which the subunits are expressed as a poly-protein and subsequently cleaved into the single subunits by TEV protease. A similar strategy has been applied e.g. in *E. coli* [18], but to our knowledge not in yeast. In the previous work, TEV-protease was part of the poly-protein, however, since we have two constructs with TEV-separated proteins, we decided to express TEV from a separate promoter. Since the TEV-based strategy was not previously tested in yeast, we decided to verify the cleavage of the target proteins into the expected sizes. We included a V5-tag on one protein from each construct (including the Sadh) for verification by western blotting (Figure 2). As shown in Figure 3, bands of sizes 20 kDa and 15 kDa, corresponding to predicted sizes for the third subunit of the diol dehydratase (pduE) and the second subunit of the activator (pduH), were both recognized on the membrane. This provided evidence for functional TEV protease expression. Comparison of the results with the control sample from a strain not carrying the dehydratase and Sadh constructs (strain *YSH6.142-3D*), showed that the recognized bands were exclusive to the strain *yPG-3*, holding the entire set of TEV expression system (Figure 3 - column A). However, intermediates of the protease cutting were also evident as three bands with approximate sizes of 110, 80 and 45 kDa which were likely to represent uncut fractions of pduCDE, pduGH and pduDE, respectively (Figure 3 - column B). The heterologous secondary alcohol dehydrogenase (Sadh – tagged with V5 epitope) appeared on the membrane as well with an approximate size of 38 KDa. There was also an unspecific band of approximately 40 kDa on the membrane which was common between the test sample and the control. The identity of this protein is unknown. We conclude that the TEV-cleavage based expression could be used for our proteins.

**Figure 4:**
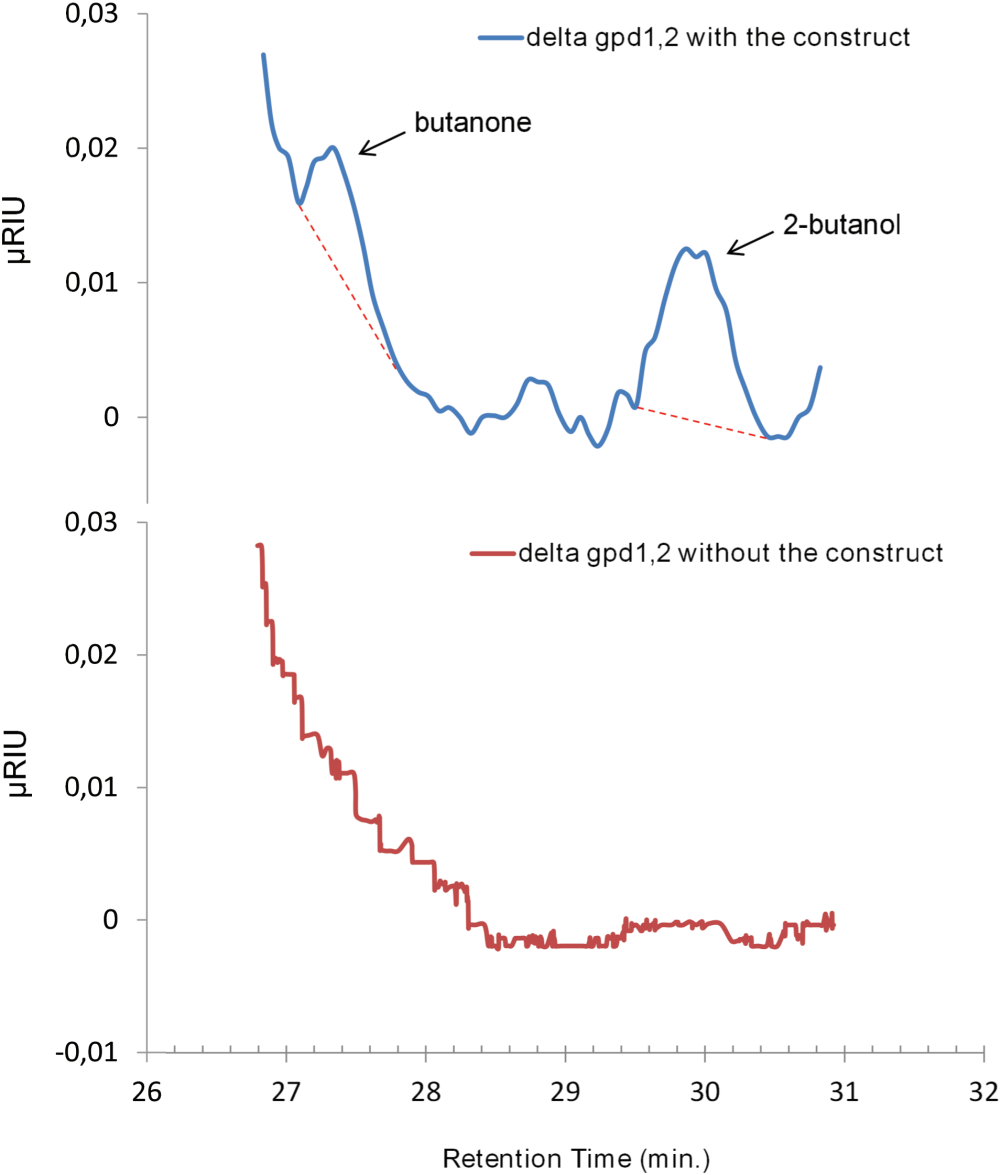
Comparison of HPLC results for both the control and constructed strain. The graph is zoomed in to the time span where 2-butanol and 2-butanone are known to appear. The base-line used to calculate the amount of butanone and butanol is indicated as a dashed line.

Correct cleavage does not necessarily imply correct folding and subunit association. An experiment was therefore performed in which the constructed strain carrying the diol-dehydratase and Sadh-constructs (*yPG-3*) were provided with 3 g/L *meso*-2,3-butanediol and 1 g/L Ado-Cbl to check its capability of producing 2-butanol. Under aerobic conditions, neither butanol nor butanone was detected, which was as expected for butanol, but somewhat surprising for butanone. However, we reasoned that this might be due to a lack of NADH for the SADH-step, since this was previously shown to require a high level of NADH (see above). We therefore cultivated the *Δgpd1,2* strain with the butanol synthetic constructs (*yPG-3*) under anaerobic condition which would require the cell to exploit the introduced 2-butanol pathway in order to meet its redox balance needs. Under these conditions and with provision of *meso*-2,3-butanediol and 1 g/L Ado-Cbl, formation of 2-butanol, equivalent to 4±0.2 mg/L, as well as 2±0.1 mg/L of butanone was obtained (Figure 4). These compounds were not produced in the strain lacking the *pduCDE* and *pduGH* gene constructs (*YSH6.142-3D*). Glucose was consumed within 24 hours while 2-butanol was detected after 40 hours with a slight increase in concentration up to a maximum of 4 mg/L after 66 hours. We also constructed the strain *yPG-4*, a *Δgpd1,2* strain with the same heterologous genes except that the *SacI*-fragment encoding the TEV protease was removed from the expression sequence (Figure 2). Consistent with the prediction that TEV-cleavage is essential for protein complex formation and function, no 2-butanol or butanone was produced when this strain was fed with *meso*-2,3-butanediol and Ado-Cbl, under anaerobic conditions.

Two other potential substrates for the diol-dehydratase (racemic 2,3-butanediol and acetoin) were also tested in the *Δgpd1,2* strain with the butanol synthetic constructs (*yPG-3*) under anaerobic conditions, but this did not lead to any 2-butanol production. This is in line with the characterization of the dehydratase in some *Lactobacillus* strains as specific for *meso*-2,3-butanediol [8], [29]. The importance of the redox burden was further manifested by the fact that a *BY4742* strain with entire set of 2-butanol heterologous genes (strain *yPG-1*) failed to produce 2-butanol from any of the substrates (*meso*-2,3-butanediol, racemic 2,3-butanediol and acetoin). This was despite the fact that the expected bands corresponding to cleaved pduH, pduE and Sadh were detected (data not shown). This argues strongly for the need for NADH surplus generated in the *Δgpd1,2* strain anaerobically.

The enzyme mediating the dehydratase activity requires Ado-Cbl, the production of which is not present in *S. cerevisiae.* We believe the availability of this co-factor to be the main limiting factor for 2-butanol production in this study. The substrate *meso*-2,3-butanediol was provided in excess and only a small amount of its consumption was converted to 2-butanol. Based on the amount of Ado-Cbl supplied to the medium (1 g/L), the theoretical yield for 2-butanol would be about 46 mg/L. Considering that the total amount of 2-butanol and butanone produced together, i.e. 6 mg/L, the conversion efficiency in this study was about 13%. At the end of the experiment the medium was still strongly red which indicated that a large part of the Ado-Cbl was still present in the medium.

Therefore to have a successful butanol production this strain would require expression of the B_12_ synthesizing and regenerating metabolism [30]. However, this would involve introduction of a pathway of approximately 20 proteins [31], making it a very challenging project. Thus, even though 2-butanol will most likely not in the near future be produced via a B_12_-dependent pathway in yeast, our study has proven that the enzyme-expressing constructs are functional and could be introduced into a host organism capable of B_12_-synthesis. An alternative in yeast could be to use a B_12_ independent diol dehydratase, e.g. from *Clostridium butyricum* [32] or *Roseburia inulirivorans* [33] and we are therefore currently exploring this possibility. Furthermore, the TEV-protease cleavage based expression system applied in this study provides a useful technique for heterologous protein expression which can be applied to various microbial engineering objectives. The advantage of this approach is that the protein subunits will be expressed at stoichiometric equal levels, and also to the same physical location.

## Supporting Information

Text S1 **Sequences of the constructs.** Sequences of the PCR-fragment used to integrate construct of *pduGH* at a locus 1000 bp downstream of the *DAK2* stop-codon and plasmid constructs for *SADH* and *pduCDE e*xpression. The integrative construct and *SADH* plasmid have a *TDH3* promoter and *pduCDE* plasmid has a *TPI1* promoter. All the constructs have a 3′UTR/terminator region from *ADH1*.
(DOCX)
