# Supplementary material for: 2-Butanol and Butanone Production in Saccharomyces cerevisiae through Combination of a B12 Dependent Dehydratase and a Secondary Alcohol Dehydrogenase Using a TEV-Based Expression System
Source: PLoS One. 2014 Jul 23;9(7):e102774. doi: 10.1371/journal.pone.0102774 (PMC4108354; doi:10.1371/journal.pone.0102774)
Supplement: Text S1 — Sequences of the constructs. Sequences of the PCR-fragment used to integrate construct of pduGH at a locus 1000 bp downstream of the DAK2 stop-codon and plasmid constructs for SADH and pduCDE expression. The integrative construct and SADH plasmid have a TDH3 promoter and pduCDE plasmid has a TPI1 promoter. All the constructs have a 3′UTR/terminator region from ADH1. (DOCX) [file pone.0102774.s001.docx]

Color code:

(green PduC, blue PduD, purple PduE, red TEV-cleavage sites, orange TPI1-promoter,TDH3-promoter, brown ADH1-terminator, grey RPL18B-3'UTR/RPS19B-promotor, light blue TEV-protease, SalI-flanked V5-tag in magenta, pink DAK2 homology site)

**Sequence of the *pduGH* construct: (PCR fragment):**

TTCATGCATCTAAGAAATCAACCTATATCAACAGATTTCAATAATTACTCTAAACTTATGCTGTAACTTAGAAAGTAACCAGCCTGTGTTGACTGATTGAGTTGCGTATTAACTGCGCCTAGTCATTTCAACACTTATAATTTGCTTCAGCTTAAGTGTGGTTCATCTTTTTTTTTCTGGAAACTTTGCATGCCCTCAAAGTCGACCTGCTGTAACCCGTACATGCCCAAAATAGGGGGCGGGTTACACAGAATATATAACATCGTAGGTGTCTGGGTGAACAGTTTATTCCTGGCATCCACTAAATATAATGGAGCCCGCTTTTTAAGCTGGCATCCAGAAAAAAAAAGAATCCCAGCACCAAAATATTGTTTTCTTCACCAACCATCAGTTCATAGGTCCATTCTCTTAGCGCAACTACAGAGAACAGGGGCACAAACAGGCAAAAAACGGGCACAACCTCAATGGAGTGATGCAACCTGCCTGGAGTAAATGATGACACAAGGCAATTGACCCACGCATGTATCTATCTCATTTTCTTACACCTTCTATTACCTTCTGCTCTCTCTGATTTGGAAAAAGCTGAAAAAAAAGGTTGAAACCAGTTCCCTGAAATTATTCCCCTACTTGACTAATAAGTATATAAAGACGGTAGGTATTGATTGTAATTCTGTAAATCTATTTCTTAAACTTCTTAAATTCTACTTTTATAGTTAGTCTTTTTTTTAGTTTTAAAACACCAAGAACTTAGTTTCGAATAAACACACATAAACAAACAAAGGATCCATGGCTACTGAAAAAGTTATTGGTGTTGATATTGGTAATTCTTCTACTGAAGTTGCTTTGGCTGATGTTGCTGATAATGGTACTATTAATTTCATTGGTTCTGGTATTGCTCCAACTACTGGTATTAAAGGTACTAAACAAAATTTGGTTGGTATTAGAGATTCTATTAATCAAGTTTTGAATAAAGCTAATTTGACTATTAATGATATTGATTTGATTAGAATTAATGAAGCTACTCCAGTTATTGGTGATGTTGCTATGGAAACTATTACTGAAACTGTTGTTACTGAATCTACTATGATTGGTCATAATCCAGATACTCCAGGTGGTATTGGTACTGGTGCTGGTATTACTGTTAGATTGTTGGATTTGGTTAAAAAAACTGATAAATCTCAAAATTATATTGTTGTTGTTCCAAAAGATATTGATTTCGAAGATGTTGCTAAATTGATTAATGCTTATGTTGCTTCTGGTTATAAAATTACTGCTGCTATTTTGAAAAATGATGATGGTGTTTTGGTTGATAATAGATTGAATAAATCTATTCCAATTGTTGATGAAGTTGCTATGATTGATAAAGTTCCATTGAATATGTTGGCTGCTGTTGAAGTTGCTGGTCCAGGTCAAGTTATTTCTCAATTGTCTAATCCATATGGTATTGCTACTTTGTTCGGTTTGAATCCAGAAGAAACTAAAAATATTGTTCCAGTTTCTAGAGCTTTGATTGGTAATAGATCTGCTGTTGTTATTAAAACTCCAGCTGGTGATGTTAAAGCTAGAGTTATTCCAGCTGGTAATATTATTATTAATGGTGATACTGGTAAAGAAGAAGTTGGTGTTTCTGAAGGTGCTGATGCTATTATGAAAAGAATTTCTTCTTTCAGACATATTAATGATATTACTGGTGAATCTGGTACTAATGTTGGTGGTATGTTGGAAAATGTTAGACAAACTATGGCTGATTTGACTGGTAAAAAAAATTCTGAAATTGCTATTCAAGATTTGTTGGCTGTTGATACTCAAGTTCCAGTTGAAGTTAGAGGTGGTTTGGCTGGTGAATTCTCTAATGAATCTGCTGTTGGTATTGCTGCTATGGTTAAATCTGATCATTTGCAAATGGAAGTTATTGCTAAATTGATTGAAGATGAATTCCATACTAAAGTTGAAATTGGTGGTGCTGAAGTTGAATCTGCTATTAGAGGTGCTTTGACTACTCCAGGTACTGATAAACCAATTGCTATTTTGGATTTGGGTGCTGGTTCTACTGATGCTTCTATTATTAATAAAGAAAATCAAACTGTTGCTATTCATTTGGCTGGTGCTGGTGATATGGTTACTATGATTATTAATTCTGAATTGGGTTTGAATGATATTCATTTGGCTGAAGATATTAAAAGATATCCATTGGCTAAAGTTGAAAATTTGTTCCAAATTAGACATGAAGATGGTTCTGTTCAATTCTTCGAAGATCCATTGCCATCTTCTTTGTTCGCTAGAGTTGTTGTTATTAAACCAGATGGTTATGAACCAGTTACTGGTAATCCATCTATTGAAAAAATTAAATTGGTTAGACAATCTGCTAAAAAAAGAGTTTTCGTTACTAATGCTTTGAGAGCTTTGAAATATGTTTCTCCAACTGGTAATATTAGAGATATTCCATTCGTTGTTATTGTTGGTGGTTCTGCTTTGGATTTCGAAATTCCACAATTGGTTACTGATGAATTGGCTCATTTCAATTTGGTTGCTGGTAGAGGTAATGTTAGAGGTGTTGAAGGTCCAAGAAATGCTGTTGCTACTGGTTTGATTTTGAGATATGGTGAAGAAAGAAGAAAACAATATGAACAAGAAAATTTGTATTTCCAAGGTATGGCTAAAAAAGAAGGTTCTAATATGAATAATGATTCTGAAAGACCATCTATTATTGTTGGTGTTGAAAATGGTACTGCTATTCCACAAAATGCTGCTCCATTGTTCAATGGTATTGAAGAAGAACAAATTCCAGTTGCTGTTAGAGAAATTGATATTGATAATGTTGTTTCTAGAGCTTATCAATCTGCTTTGGCTTCTAGATTGTCTGTTGGTATTGCTTTCGATGGTGATAGATTCATTGTTCATTATAAAAATTTGAAAGAAAATAAACCATTGTTCGATAAAACTATTTCTGATGGTAAACAATTGAGAGTTTTGGGTGCTAATGCTGCTAGATTGGTTAAAGGTATTCCATTCAAAGAAATGGTTAATAGACTCGAGGGTAAACCAATTCCAAATCCATTGTTGGGTTTGGATTCTACTCTCGAGTAAGGCGCGCCACTTCTAAATAAGCGAATTTCTTATGATTTATGATTTTTATTATTAAATAAGTTATAAAAAAAATAAGTGTATACAAATTTTAAAGTGACTCTTAGGTTTTAAAACGAAAATTCTTATTCTTGAGTAACTCTTTCCTGTAGGTCAGGTTGCTTTCTCAGGTATAGTATGAGGTCGCTCTTATTGACCACACCTCTACCGGCAGATCCGCTAGGGATAACAGGGTAATATGAGCTCATAACTTCGTATAATGTATGCTATACGAAGTTATGCGGCCGCTAGGTCTAGAGATCTGTTTAGCTTGCCTCGTCCCCGCCGGGTCACCCGGCCAGCGACATGGAGGCCCAGAATACCCTCCTTGACAGTCTTGACGTGCGCAGCTCAGGGGCATGATGTGACTGTCGCCCGTACATTTAGCCCATACATCCCCATGTATAATCATTTGCATCCATACATTTTGATGGCCGCACGGCGCGAAGCAAAAATTACGGCTCCTCGCTGCAGACCTGCGAGCAGGGAAACGCTCCCCTCACAGACGCGTTGAATTGTCCCCACGCCGCGCCCCTGTAGAGAAATATAAAAGGTTAGGATTTGCCACTGAGGTTCTTCTTTCATATACTTCCTTTTAAAATCTTGCTAGGATACAGTTCTCACATCACATCCGAACATAAACAACCATGGGTAAGGAAAAGACTCACGTTTCGAGGCCGCGATTAAATTCCAACATGGATGCTGATTTATATGGGTATAAATGGGCTCGCGATAATGTCGGGCAATCAGGTGCGACAATCTATCGATTGTATGGGAAGCCCGATGCGCCAGAGTTGTTTCTGAAACATGGCAAAGGTAGCGTTGCCAATGATGTTACAGATGAGATGGTCAGACTAAACTGGCTGACGGAATTTATGCCTCTTCCGACCATCAAGCATTTTATCCGTACTCCTGATGATGCATGGTTACTCACCACTGCGATCCCCGGCAAAACAGCATTCCAGGTATTAGAAGAATATCCTGATTCAGGTGAAAATATTGTTGATGCGCTGGCAGTGTTCCTGCGCCGGTTGCATTCGATTCCTGTTTGTAATTGTCCTTTTAACAGCGATCGCGTATTTCGTCTCGCTCAGGCGCAATCACGAATGAATAACGGTTTGGTTGATGCGAGTGATTTTGATGACGAGCGTAATGGCTGGCCTGTTGAACAAGTCTGGAAAGAAATGCATAAGCTTTTGCCATTCTCACCGGATTCAGTCGTCACTCATGGTGATTTCTCACTTGATAACCTTATTTTTGACGAGGGGAAATTAATAGGTTGTATTGATGTTGGACGAGTCGGAATCGCAGACCGATACCAGGATCTTGCCATCCTATGGAACTGCCTCGGTGAGTTTTCTCCTTCATTACAGAAACGGCTTTTTCAAAAATATGGTATTGATAATCCTGATATGAATAAATTGCAGTTTCATTTGATGCTCGATGAGTTTTTCTAATCAGTACTGACAATAAAAAGATTCTTGTTTTCAAGAACTTGTCATTTGTATAGTTTTTTTATATTGTAGTTGTTCTATTTTAATCAAATGTTAGCGTGATTTATATTTTTTTTCGCCTCGACATCATCTGCCCAGATGCGAAGTTAAGTGCGCAGAAAGTAATATCATGCGTCAATCGTATGTGAATGCTGGTCGCTATACTGCTGTCGATTCGATACTAACGCCGCCATCCAGTGTCGAAAACGAGCTTTCGAGAACCCTTAATGCGGCCGCATAACTTCGTATAATGTATGCTATACGAAGTTATGCATGAGTAGTTAGTTATCTTTTTGACAATGATCTCTTTTGAAAATATCTACTGTAGATTTGCATGGACGCACGTCGCCCATACGCCAAACTTTGGCAATGATACTCGTTATTCGTAATATCAGTCCGTCAAGGTGCTGTGATTTCTCTATTTTATATTGCCTATTATTTTTTCAAATGATTTGAGCCGTTTTAAATTGA

**Sequence of the *pduCDE* construct:**

CTCGAGATTTAAACTGTGAGGACCTTAATACATTCAGACACTTCTGCGGTATCACCCTACTTATTCCCTTCGAGATTATATCTAGGAACCCATCAGGTTGGTGGAAGATTACCCGTTCTAAGACTTTTCAGCTTCCTCTATTGATGTTACACCTGGACACCCCTTTTCTGGCATCCAGTTTTTAATCTTCAGTGGCATGTGAGATTCTCCGAAATTAATTAAAGCAATCACACAATTCTCTCGGATACCACCTCGGTTGAAACTGACAGGTGGTTTGTTACGCATGCTAATGCAAAGGAGCCTATATACCTTTGGCTCGGCTGCTGTAACAGGGAATATAAAGGGCAGCATAATTTAGGAGTTTAGTGAACTTGCAACATTTACTATTTTCCCTTCTTACGTAAATATTTTTCTTTTTAATTCTAAATCAATCTTTTTCAATTTTTTGTTTGTATTCTTTTCTTGCTTAAATCTATAACTACAAAAAACACATACATAAACTAAAAGGCGCGCCATGAAAAGACAAAAAAGATTCGAAGAATTGGAAAAAAGACCAATTCATCAAGATACTTTCGTTAAAGAATGGCCAGAAGAAGGTTTCGTTGCTATGATGGGTCCAAATGATCCAAAACCATCTGTTAAAGTTGAAAATGGTAAAATTGTTGAAATGGATGGTAAAAAATTGGAAGATTTCGATTTGATTGATTTGTATATTGCTAAATATGGTATTAATATTGATAATGTTGAAAAAGTTATGAATATGGATTCTACTAAAATTGCTAGAATGTTGGTTGATCCAAATGTTTCTAGAGAATCTATTATTGAAATTACTTCTGCTTTGACTCCAGCTAAAGCTGAAGAAATTATTTCTAAATTGGATTTCGGTGAAATGATTATGGCTATTAAAAAAATGAGACCAAGAAGAAAACCAGATAATCAATGTCATGTTACTAATACTGTTGATAATCCAGTTCAAATTGCTGCTGATGCTGCTGATGCTGCTTTGAGAGGTTTCCCAGAACAAGAAACTACTACTGCTGTTGCTAGATATGCTCCATTCAATGCTATTTCTATTTTGATTGGTGCTCAAACTGGTAGACCAGGTGTTTTGACTCAATGTTCTGTTGAAGAAGCTACTGAATTGCAATTGGGTATGAGAGGTTTCACTGCTTATGCTGAAACTATTTCTGTTTATGGTACTGATAGAGTTTTCACTGATGGTGATGATACTCCATGGTCTAAAGGTTTCTTGGCTTCTTGTTATGCTTCTAGAGGTTTGAAAATGAGATTCACTTCTGGTGCTGGTTCTGAAGTTTTGATGGGTTATCCAGAAGGTAAATCTATGTTGTATTTGGAAGCTAGATGTATTTTGTTGACTAAAGCTTCTGGTGTTCAAGGTTTGCAAAATGGTGCTGTTTCTTGTATTGAAATTCCAGGTGCTGTTCCAAATGGTATTAGAGAAGTTTTGGGTGAAAATTTGTTGTGTATGATGTGTGATATTGAATGTGCTTCTGGTTGTGATCAAGCTTATTCTCATTCTGATATGAGAAGAACTGAAAGATTCATTGGTCAATTCATTGCTGGTACTGATTATATTAATTCTGGTTATTCTTCTACTCCAAATTATGATAATACTTTCGCTGGTTCTAATACTGATGCTATGGATTATGATGATATGTATGTTATGGAAAGAGATTTGGGTCAATATTATGGTATTCATCCAGTTCAAGAAGAAACTATTATTAAAGCTAGAAATAAAGCTGCTAAAGCTTTGCAAGCTGTTTTCGAAGATTTGGGTTTGCCAAAAATTACTGATGAAGAAGTTGAAGCTGCTACTTATGCTAATACTCATGATGATATGCCAAAAAGAGATATGGTTGCTGATATGAAAGCTGCTCAAGATATGATGGATAGAGGTATTACTGCTGTTGATATTATTAAAGCTTTGTATAATCATGGTTTCAAAGATGTTGCTGAAGCTGTTTTGAATTTGCAAAAACAAAAAGTTGTTGGTGATTATTTGCAAACTTCTTCTATTTTCGATAAAGATTGGAATATTACTTCTGCTGTTAATGATGGTAATGATTATCAAGGTCCAGGTACTGGTTATAGATTGTATGAAGATAAAGAAGAATGGGATAGAATTAAAGATTTGCCATTCGCTTTGGATCCAGAACATTTGGAATTGGACGTCGAAAATTTGTATTTCCAAGGTATGGCTGATATTGATGAAAATTTGTTGAGAAAAATTGTTAAAGAAGTTTTGAATGAAACTAATCAAATTGATACTAAAATTAATTTCGATAAAGAAAATAATTCTACTGCTACTGCTACTGAAGAAGTTCAACAACCAAATTCTAAAGCTGTTCCAGAAAAAAAATTGGATTGGTTCCAACCAATTGGTGAAGCTAAACCAGGTTATTCTAAAGATGAAGTTGTTATTGCTGTTGGTCCAGCTTTCGCTACTGTTTTGGATAAAACTGAAACTGGTATTCCACATAAAGAAGTTTTGAGACAAGTTATTGCTGGTATTGAAGAAGAAGGTTTGAAAGCTAGAGTTGTTAAAGTTTATAGATCTTCTGATGTTGCTTTCTGTGCTGTTCAAGGTGATCATTTGTCTGGTTCTGGTATTGCTATTGGTATTCAATCTAAAGGTACTACTGTTATTCATCAAAAAGATCAAGATCCATTGGGTAATTTGGAATTGTTCCCACAAGCTCCAGTTTTGACTCCAGAAACTTTCAGAGCTATTGGTAAAAATGCTGCTATGTATGCTAAAGGTGAATCTCCAGAACCAGTTCCAGCTAAAAATGATCAATTGGCTAGAATTCATTATCAAGCTATTTCTGCTATTATGCATATTAGAGAAACTCATCAAGTTGTTGTTGGTAAACCAGAAGAAGAAATTAAAGTTACTTTCGATGCTAGCGAAAATTTGTATTTCCAAGGTATGTCTGAAGTTGATGATTTGGTTGCTAAAATTATGGCTCAAATGGGTAATTCTTCTTCTTCTGATTCTTCTACTTCTGCTACTTCTACTAATAATGGTAAAGAAATGACTGCTGATGATTATCCATTGTATCAAAAACATAGAGATTTGGTTAAAACTCCATCTGGTAAAAAATTGGATGATATTACTTTGCAAAAAGTTGTTAATGATCAAGTTAATCCAAAAGAATTGAGAATTACTCCAGAAGCTTTGAAATTGCAAGGTGAAATTGCTGCTAATGCTGGTAGACCAGCTATTCAAAAAAATTTGCAAAGAGCTGCTGAATTGACTAGAGTTCCAGATGAAAGAGTTTTGCAAATGTATGATGCTTTGAGACCATTCAGATCTACTAAACAAGAATTGTTGGATATTGCTAATGAATTGAGAGATAAATATCATGCTGAAGTTTGTGCTGCTTGGTTCGAAGAAGCTGCTGATTATTATGAATCTAGAAAAAAATTGAAAGGTGATAATGTCGACGGTAAACCAATTCCAAATCCATTGTTGGGTTTGGATTCTACTGTCGACTAATCTAGTATGGTTTGAAACCTTACAATTTTTCTTCTTTGTTCCTTTTTCCTTGTTTCAGTGTATATTAGGTTGGGAAAGAGGGATTTTTCCATACCATATGACTGACTACAATATATACATGTATAATAACTTCATAATCTAAACCTATCAGTTCAGTATCAAGTCAGCTATTCCGCCCTATGCATAAACCTACAAACTATCATTCACACACTTTTCCCATTTTTTTTCAATACTACTTTACATCCGAACATTTTAGCAACCCACACCATATACCTTTGGTGCACTATTGATTTTCTTCCTGATGTCAGCTTTTTGTGCTTTGACAAAAAAATCGCGTCTACGTCCGTCCGTTCTCCCTGAATAAATTAGGCGCGTTTGAGCCCAGCAGGACGGAGCTCTAGTGACAAGCCCTGGTGTTTGGTGAGGTTTTGCACATTGCTGTTCTTTCTACTGTATTGAGATCTCCAGTTTACGGCTCCCTGGGAGCCACCCGTAACGCGGTTGGTGTGCCCATTTCAATAAGCGAACATTAGTGAAGATACAATCGTTAAAATGGACTAATGAAATTTTAAAGTGGGATTTTTGTGAATATTGACAACAAAGGTATAGAACCAAAGATAATAAAGATGGGTGAATCTTTGTTCAAAGGTCCAAGAGATTATAATCCAATTTCTTCTACTATTTGTCATTTGACTAATGAATCTGATGGTCATACTACTTCTTTGTATGGTATTGGTTTCGGTCCATTCATTATTACTAATAAACATTTGTTCAGAAGAAATAATGGTACTTTGTTGGTTCAATCTTTGCATGGTGTTTTCAAAGTTAAAAATACTACTACTTTGCAACAACATTTGATTGATGGTAGAGATATGATTATTATTAGAATGCCAAAAGATTTCCCACCATTCCCACAAAAATTGAAATTCAGAGAACCACAAAGAGAAGAAAGAATTTGTTTGGTTACTACTAATTTCCAAACTAAATCTATGTCTTCTATGGTTTCTGATACTTCTTGTACTTTCCCATCTTCTGATGGTATTTTCTGGAAACATTGGATTCAAACTAAAGATGGTCAATGTGGTTCTCCATTGGTTTCTACTAGAGATGGTTTCATTGTTGGTATTCATTCTGCTTCTAATTTCACTAATACTAATAATTATTTCACTTCTGTTCCAAAAAATTTCATGGAATTGTTGACTAATCAAGAAGCTCAACAATGGGTTTCTGGTTGGAGATTGAATGCTGATTCTGTTTTGTGGGGTGGTCATAAAGTTTTCATGGTTAAACCAGAAGAACCATTCCAACCAGTTAAAGAAGCTACTCAATTGATGAATAGAAGAAGAAGAAGAATCGATTAGACTTCTAAATAAGCGAATTTCTTATGATTTATGATTTTTATTATTAAATAAGTTATAAAAAAAATAAGTGTATACAAATTTTAAAGTGACTCTTAGGTTTTAAAACGAAAATTCTTATTCTTGAGTAACTCTTTCCTGTAGGTCAGGTTGCTTTCTCAGGTATAGTATGAGGTCGCTCTTATTGACCACACCTCTACCGGCAGATCCGCTAGGGATAACAGGGTAATATGGCGCCGCGGCCGC

**Sequence of the *SADH* construct:**

CTCGAGGAACAGTTTATTCCTGGCATCCACTAAATATAATGGAGCCCGCTTTTTAAGCTGGCATCCAGAAAAAAAAAGAATCCCAGCACCAAAATATTGTTTTCTTCACCAACCATCAGTTCATAGGTCCATTCTCTTAGCGCAACTACAGAGAACAGGGGCACAAACAGGCAAAAAACGGGCACAACCTCAATGGAGTGATGCAACCTGCCTGGAGTAAATGATGACACAAGGCAATTGACCCACGCATGTATCTATCTCATTTTCTTACACCTTCTATTACCTTCTGCTCTCTCTGATTTGGAAAAAGCTGAAAAAAAAGGTTGAAACCAGTTCCCTGAAATTATTCCCCTACTTGACTAATAAGTATATAAAGACGGTAGGTATTGATTGTAATTCTGTAAATCTATTTCTTAAACTTCTTAAATTCTACTTTTATAGTTAGTCTTTTTTTTAGTTTTAAAACACCAAGAACTTAGTTTCGAATAAACACACATAAACAAACAGGATCCATGAAAGCTTTGGTTTTCCATGGTCCAGGTCAAAAAGCTTGGGAAGATGTTCCAGATCCAAAATTGTCTGAACCAACTGATGTTATTGTTAAAATGGATACTACTACTATTTGTGGTACTGATTTGCATATTTTGAAAGGTGATGTTCCAGCTGTTACTCCAGGTAGAATTTTGGGTCATGAAGGTGTTGGTACTATTACTGAAGTTGGTGATGCTGTTACTACTTTGGCTGTTGGTGATCAAGTTATTTTGTCTTGTATTTCTTCTTGTGGTAAATGTTCTTTCTGTAAACAAGGTGTTTATTCTCATTGTTTGGGTTCTGAAGGTACTTCTGGTATTGGTTGGATTTTCGGTCATTTGATTGATGGTACTCAAGCTGAATATGTTAGAGTTCCATATGCTGAAAATTCTGTTCATAAATTGCCATCTGCTGTTTCTCCAGAACAAGGTACTTTGTTGTCTGATATTTTGCCAACTGGTCATGAAATTGGTGTTAGACATGGTGCTGTTAAACCAGGTGATGTTGTTGCTGTTATTGGTACTGGTCCAGTTGGTTTGGCTGCTATTGCTACTGCTGGTTTGTATGGTCCATCTAGAGTTATTGCTGTTGATATTGATGCTAATAGAGTTGAACAAGCTAGAAGATTCGGTGCTACTGATGGTGTTGTTTCTACTTCTTCTGGTTGGAGAGAAGAAATTTTGGCTATGACTGATGGTTTGGGTGTTGATGTTGCTATTGAAGCTGTTGGTATTCCAGATACTTTCCAAATGTGTTTGGATATTGTTAGACCAGCTGGTCATGTTGCTAATGTTGGTGTTCATGGTAAACCAGTTGAATTGCCAATTCAAGATTTGTGGATTTCTAATATTGTTATGTCTATGGGTTTGGTTAATACTAATACTTTGGGTACTTTGTTGAAATTGGTTGCTCAAAGAAGAATTGATCCAGAACCATTCATTTCTCATAGATTCGGTTTGGGTGAAATTATTGATGCTTATGATGTTTTCTCTAGAGCTGCTGAAACTAAAGCTTTGAAAGTTATTATGTCTGCTGTCGACGGTAAACCAATTCCAAATCCATTGTTGGGTTTGGATTCTACTGTCGACTAGGGCGCGCCACTTCTAAATAAGCGAATTTCTTATGATTTATGATTTTTATTATTAAATAAGTTATAAAAAAAATAAGTGTATACAAATTTTAAAGTGACTCTTAGGTTTTAAAACGAAAATTCTTATTCTTGAGTAACTCTTTCCTGTAGGTCAGGTTGCTTTCTCAGGTATAGTATGAGGTCGCTCTTATTGACCACACCTCTACCGGCAGATCCGCTAGGGATAACAGGGTAATATGAGCTC
